# *Bartonella* infections are rare in blood-fed *Ixodes scapularis* and *Ixodes pacificus* ticks collected from rodents in the United States

**DOI:** 10.1186/s13071-024-06541-w

**Published:** 2024-10-29

**Authors:** Ying Bai, Lynn M. Osikowicz, Jacoby Clark, Erik Foster, Christina Parise, Sarah Maes, Rebecca J. Eisen

**Affiliations:** Division of Vector-Borne Diseases, National Center for Emerging and Zoonotic Infectious Diseases, Centers for Disease Control and Prevention, Fort Collins, CO USA

**Keywords:** *Bartonella* spp., Blood-fed, Ixodes scapularis, Ixodes pacificus, Rodents, Fleas

## Abstract

**Background:**

*Ixodes scapularis* and *Ixodes pacificus* are important vectors of multiple pathogens in the United States. However, their role in transmission of *Bartonella* spp., which are commonly reported in rodents and fleas, has been debated. Our previous investigation on *Bartonella* spp. in host-seeking *I. scapularis* and *I. pacificus* showed *Bartonella* spp. were absent in the ticks, suggesting the two species are unlikely to contribute to *Bartonella* transmission. It is unclear whether the absence of *Bartonella* spp. in the host-seeking ticks was attributable to ticks not being exposed to *Bartonella* in nature or being exposed but unable to acquire or transstadially transmit the bacterium. To assess the likelihood of exposure and acquisition, we tested *Ixodes* spp. ticks collected from rodents for *Bartonella* infections.

**Methods:**

Blood-fed *I. scapularis* ticks (*n* = 792; consisting of 645 larvae and 147 nymphs), *I. pacificus* ticks (*n* = 45, all larvae), and *Ixodes angustus* ticks (*n* = 16, consisting of 11 larvae and 5 nymphs) collected from rodents from Minnesota and Washington were tested for *Bartonella* spp. using a quadruplex polymerase chain reaction (PCR) amplicon next-generation sequencing approach that targets *Bartonella*-specific fragments on *gltA*, *ssrA*, *rpoB*, and *groEL.* In parallel, rodents and fleas collected from the same field studies were investigated to compare the differences of *Bartonella* distribution among the ticks, fleas, and rodents.

**Results:**

*Bartonella* spp. were commonly detected in rodents and fleas, with prevalence of 25.6% in rodents and 36.8% in fleas from Minnesota; 27.9% in rodents and 45.2% in fleas from Washington. Of all tested ticks, *Bartonella* DNA was detected by *gltA* in only one larval *I. scapularis* tick from Minnesota.

**Conclusions:**

The high prevalence of *Bartonella* spp. in rodents and fleas coupled with extremely low prevalence of *Bartonella* spp. in blood-fed ticks suggests that although *Ixodes* ticks commonly encounter *Bartonella* in rodents, they rarely acquire the infection through blood feeding. Notably, ticks were at various stages of feeding on rodents when they were collected. Laboratory transmission studies are needed to assess acquisition rates in fully blood-fed ticks and to assess transstadial transmission efficiency if ticks acquire *Bartonella* infections from feeding to repletion.

**Graphical Abstract:**

**Figure:**
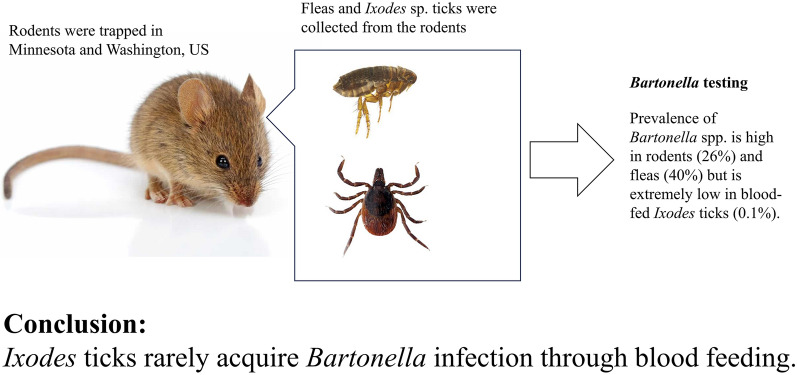

## Background

The Gram-negative *Bartonella* bacteria are widely distributed in a variety of mammalian hosts. Particularly, rodents of many species have been reported to be reservoirs for more than 30 *Bartonella* species [1–6]. Some *Bartonella* spp., such as *Bartonella elizabethae*, *B. vinsonii* subsp. *arupensis*, and *B. grahamii* have been associated with human illnesses and considered zoonotic pathogens [7–11]. Host specificity has been observed for many *Bartonella* spp. For example, *B. elizabethae* is *Rattus* rat-associated [6], while *B. vinsonii* subsp. *arupensis* is specific to *Peromyscus* sp. mice [4, 12].

*Bartonella* spp. are mainly transmitted by hematophagous arthropods, including sandflies (*Lutzomyia verrucarum*), human body lice (*Pediculus humanus corporis*), cat fleas (*Ctenocephalides felis*), and some rodent fleas [13–17]. The role of other arthropods, such as ticks, in the natural cycle of *Bartonella* spp. and the transmission of these bacteria has been debated. Molecular surveys from different regions of the world on *Bartonella* infections in host-seeking ticks, mainly *Ixodes* ticks, have reported conflicting results, with detection of *Bartonella* DNA in high or low proportions or none of the tested ticks [18–30].

The blacklegged tick (*Ixodes scapularis*) and the western blacklegged tick (*Ixodes pacificus*) are widely distributed in the eastern and far western US, respectively. These ticks frequently bite people [31] and serve as vectors of the Lyme disease spirochete and several other human pathogens [32–34]. We recently tested ~ 2600 host-seeking *I. scapularis* and *I. pacificus* nymphs and adults collected across a broad geographic region in the US for *Bartonella* spp. [35]. Consistent with some previous reports [22, 24, 29], we did not detect any *Bartonella* DNA in the ticks. Our findings suggested that *I. scapularis* and *I. pacificus* ticks are unlikely to contribute to transmission of *Bartonella* spp. These findings raised several questions. Specifically, which *Bartonella* species do *Ixodes* ticks encounter in nature? When feeding on an infected host, how efficiently do they acquire *Bartonella* infection and how efficiently are bacteria transmitted transstadially?

Previous studies on *Bartonella* spp. in ticks have mostly focused on host-seeking ticks with few studies focused on blood-fed ticks derived from animal hosts [36–43]. Most of the studies on blood-fed ticks showed low to moderate *Bartonella* prevalence in the ticks, especially *Ixodes* ticks. For example, in Poland, *Bartonella* spp. were detected in 5.2% of *Ixodes ricinus* ticks collected from deer [36]; in the UK, 1.3% of *I. ricinus* ticks collected from cats showed *Bartonella* infection [39]; a study from the US reported 13.3% of *I. scapularis* ticks being infected with *Bartonella* species based on testing of 15 ticks collected from white-footed mice (*Peromyscus leucopus*) in southern Indiana using 454 pyrosequencing technology [43].

*Peromyscus* mice, including *P. leucopus* and *P. maniculatus* are common sources of blood meals for *I. scapularis* and *I. pacificus* ticks in the US [44]. These mice are known to be infected frequently with *Bartonella* spp., specifically, *B. vinsonii* subsp. *arupensis* [12], a species that has been associated with human bartonellosis [8, 10, 11]. In the present study, we analyzed a large number of blood-fed larvae and nymphs of *I. scapularis*, *I. pacificus*, and *Ixodes angustus* collected from *P. leucopus*, *P. maniculatus*, and rodents of other species trapped in Minnesota and Washington for the presence of *Bartonella* DNA to study the dynamics of *Bartonella* infection in the ticks and as a follow-up of our previous study [35]. Meanwhile, we tested rodent blood and fleas collected from rodents to learn the status of *Bartonella* distribution and to identify which *Bartonella* spp. were circulating in our study sites.

## Methods

### Study sites and sample collections

**Fig. 1:**
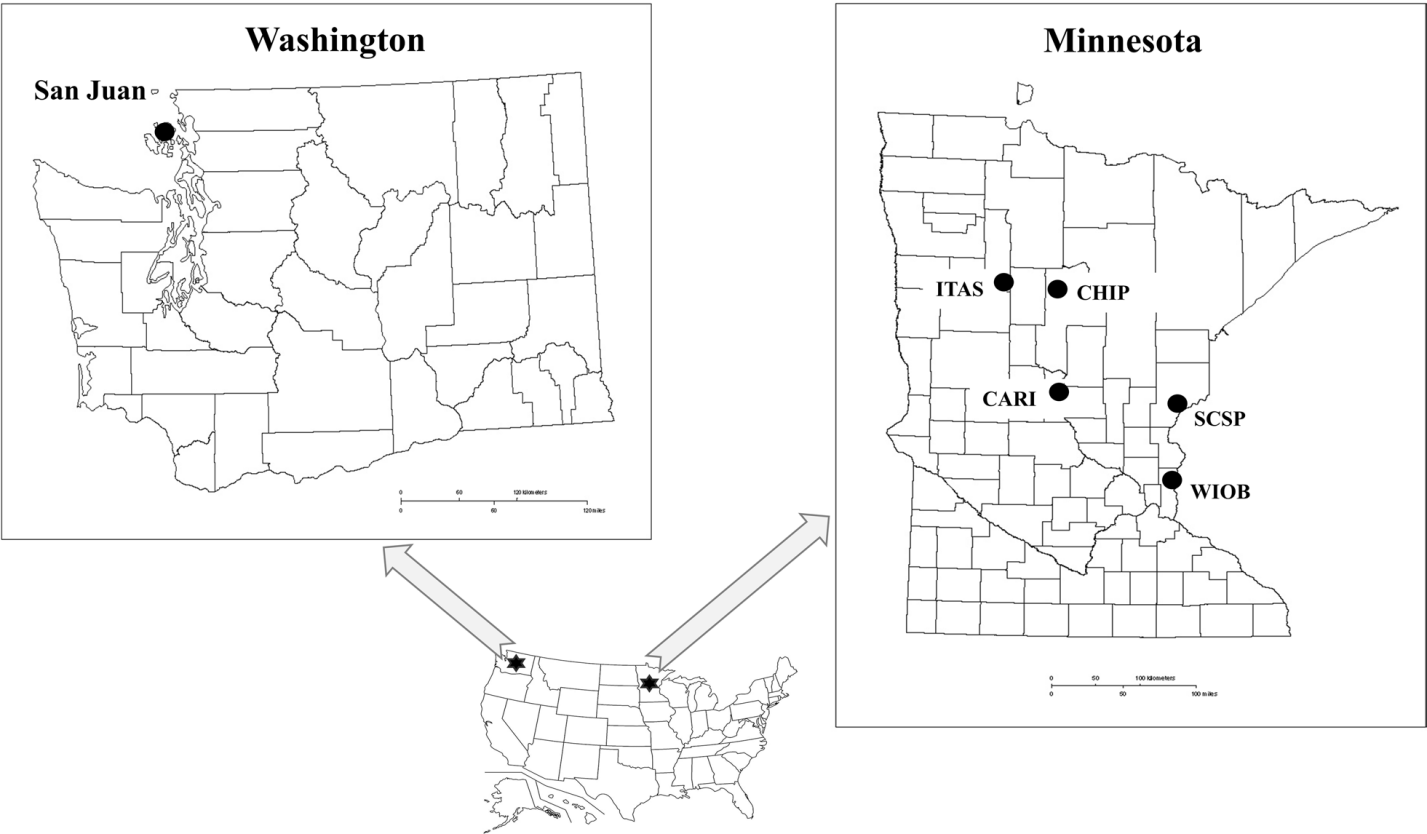
Study sites. Rodents were trapped in Camp Ripley (CARI), Chippewa National Forest (CHIP), Itasca State Park (ITAS), Saint Croix State Park (SCSP), and William O’Brien State Park (WIOB) in Minnesota, June 2016, and San Juan County, Washington, April 2019

The ticks, rodents, and fleas included in the present investigation were residuals of samples from two previous field studies conducted by CDC and other investigators at Camp Ripley (CARI), Chippewa National Forest (CHIP), Itasca State Park (ITAS), Saint Croix State Park (SCSP), and William O’Brien State Park (WIOB) in Minnesota in June 2016 and in San Juan County, Washington in April 2019 (Fig. 1). The study sites are of forested recreational settings in Minnesota and forested rural residential setting in Washington. In both studies, rodents were live-trapped using Sherman traps (H. B. Sherman Traps, Inc., Tallahassee, FL) and Tomahawk traps (Tomahawk Live Trap, Hazelhurst, WI) as previously described [45]. Rodents were identified to species morphologically using Peterson Field Guide [46] or genetically by *ctyB* using primers Pero_F and Pero_R [45], and blood was collected through submandibular venipuncture under isoflurane anesthesia, or post-mortem by cardiocentesis. Ticks and fleas were collected from the animal hosts and morphologically identified to species, sex, and life stage using taxonomic keys [47–50]. Notably, ticks were at various stages of engorgement when they were collected. All animal procedures were approved by the CDC Division of Vector-Borne Diseases (DVBD) Institutional Animal Care and Use Committee. All samples were shipped to CDC-DVBD in Fort Collins, Colorado.

DNA of rodent blood was extracted previously using the *cador* Pathogen 96 QIAcube HT Kit on the QIAcube HT (Qiagen, Chatsworth, CA) and stored at -80 ℃. Residual DNA was used for the present study; DNA of Washington ticks had been extracted previously using the MagMAX™ Pathogen Kit on the KingFisher DNA extraction system (Thermo Fisher Scientific, Waltham, MA, USA). Residual DNA was used for the present study; DNA of Minnesota ticks and all fleas from both states were freshly extracted for use in the present study using the MagMAX™ CORE Kit on the KingFisher DNA extraction system (Thermo Fisher Scientific, Waltham, MA, USA). Negative extraction controls were included to detect contaminations.

### PCR amplification, library preparation, next-generation sequencing, and bioinformatics analysis

All DNA samples, including tick DNA, flea DNA, and blood DNA, were tested for the presence of *Bartonella* spp. using a quadruplex PCR amplicon next-generation sequencing assay that targets *gltA*, *ssrA*, *rpoB*, and *groEL* using *Bartonella*-specific primers [51]. Presumptive presence of *Bartonella* DNA is defined when one target sequence has been obtained, and *Bartonella* species is confirmed when sequences of at least two targets have been obtained [51].

Detailed procedures follow those published elsewhere [35, 51]. Briefly, a primary PCR reaction containing 12.5 μl TEMPase 2 × master mix (AMPLICON, Denmark), the four pairs of *Bartonella*-specific primers (final concentration of 300 nM each), and 5 μl tick DNA was first amplified. Positive and negative controls were included in each PCR run to evaluate performance and detect contamination. Upon completion of amplification, the PCR products were purified with AMPure XP magnetic beads (Beckman Coulter, Brea, CA, USA) followed by index PCR using dual unique barcode indices (Nextera XT Index Kit V2, Illumina, San Diego, CA, USA) and then purified with MagSi-DNA allround magnetic beads (BOCA Scientific, Westwood, MA, USA). The purified products were then pooled, quantified, normalized, and denatured to generate the final library to be loaded into a MiSeq Nano v2 (500 cycles) reagent cassette (Illumina, San Diego, CA, USA) to start sequencing on an Illumina MiSeq instrument (Illumina, San Diego, CA, USA).

After sequencing was completed, raw sequences were analyzed with a custom Nextflow bioinformatics pipeline described by Osikowicz et al. [52]. Briefly, quality control analysis and primer trimming were first performed followed by error correction, paired read merging, and amplicon sequence variant (ASV) grouping. The observed ASVs were then aligned to reference sequences with the nucleotide Basic Local Alignment Search Tool (BLASTn) [53, 54]. The minimum read cut-off for a sample to be considered positive was set to 50 reads. A 95% sequence similarity and 90% minimum sequence alignment length were used to align the observed ASVs to the reference sequences. Sequences that represent different *Bartonella* species for each target were obtained from GenBank and used as reference sequences.

### Statistical analysis

Chi-square analyses were performed using the Chi Square Test Excel Function to determine whether *Bartonella* prevalences differed among the study sites in Minnesota and to compare *Bartonella* prevalence between tick-infested and tick-free rodents from both Minnesota and Washington.

## Results

### Summary of rodent and ectoparasite samples

In Minnesota, blood was obtained from 215 individual rodents, with 41, 57, 25, 61, and 31 from site CARI, CHIP, ITAS, SCSP, and WIOB, respectively (Table 1). The rodents belonged to nine species with *P. leucuous* as the most prevalent species (58.1%; 125/215). Other common species included *Clethrionomys gapperi* (17.2%; 37/215) and *P. maniculatus* (9.8%; 21/215) (Table 2). Of all rodents, 159 (74%) were infested with ticks. A total of 792 ticks consisting of 645 larvae and 147 nymphs were tested in the present study (Table 3). All ticks collected in Minnesota were identified as *I. scapularis*. Among the ticks, 140 were collected from 37 *Bartonella*-infected rodents (see the following section “*Bartonella* spp. in rodents, fleas, and ticks from Minnesota” for data on *Bartonella*-infected rodents). Fleas collected from rodents from site SCSP were included in this study, with a total of 68 fleas of six species collected from 30 rodents (Table 4).

**Table 1:** *Bartonella* distribution in rodents at different site in Minnesota (June 2016)

Site	No. rodents tested	No. *Bartonella*-positive rodents	*Bartonella* prevalence (%)
Camp Ripley (CARI)	41	5	12.2
Chippewa National Forest (CHIP)	57	19	33.3
Itasca State Park (ITAS)	25	8	32
Saint Croix State Park (SCSP)	61	18	29.5
William O’Brien State Park (WIOB)	31	5	16.1
Total	215	55	25.6

**Table 2:** *Bartonella* distribution in rodents of different species captured in Minnesota (June 2016) and Washington (April 2019)

Rodent species	Collection state	No. tested	Proportion (%)	*Bartonella* testing		*Bartonella* species		
				No. positive	Prevalence (%)	*B. vinsonii* subsp. *vinsonii*	*B. vinsonii* subsp. *arupensis*	*B. grahamii*
*Clethrionomys gapperi*	Minnesota	37	17.2	12	32.4	3		9
*Glaucomys volans*	Minnesota	3	1.4	0	0			
*Microtus pennsylvanicus*	Minnesota	1	0.5	0	0			
*Peromyscus leucopus*^a^	Minnesota	125	58.1	35	28		35	
*Peromyscus maniculatus*^a^	Minnesota	21	9.8	8	38.1		8	
*Sciurus carolinensis*	Minnesota	1	0.5	0	0			
*Tamias striatus*	Minnesota	19	8.8	0	0			
*Tamiasciurus hudsonicus*	Minnesota	4	1.9	0	0			
*Zapus hudsonicus*	Minnesota	4	1.9	0	0			
Subtotal	Minnesota	215	100	55	25.6			
*Peromyscus maniculatus*	Washington	43	100	12	27.9		12	

^a^*Peromyscus leucopus* and *Peromyscus maniculatus* collected in Minnesota were genetically differentiated by *ctyB* using primers Pero_F and Pero_R [45]

**Table 3:** *Bartonella* infections in blood-fed *Ixodes* ticks collected from rodents captured in Minnesota (June 2016) and Washington (April 2019)

Tick species	Collection state/site	From all rodents						From *Bartonella*-infected rodents				
		Life stage		*Bartonella* testing		Prevalence (%)	Life stage			*Bartonella* testing		Prevalence (%)
		Larva	Nymph	No. tested	No. positive		Larva		Nymph	No. tested	No. positive	
*Ixodes scapulars*	Minnesota/CARI	224	107	331	1^a^	0.3	24		2	26	1^a^	3.8
*Ixodes scapulars*	Minnesota/CHIP	75	6	81	0	0	21		0	21	0	0
*Ixodes scapulars*	Minnesota/ITAS	116	6	122	0	0	57		3	60	0	0
*Ixodes scapulars*	Minnesota/SCSP	146	18	164	0	0	29		0	29	0	0
*Ixodes scapulars*	Minnesota/WIOB	84	10	94	0	0	4		0	4	0	0
Subtotal	Minnesota	645	147	792	1^a^	0.1	135		5	140	1^a^	0.7
*Ixodes pacificus*	Washington/San Juan	45	0	45	0	0	10		1	11	0	0
*Ixodes angustus*	Washington/San Juan	11	5	16	0	0	5		2	7	0	0
Subtotal	Washington	56	5	61	0	0	15		3	18	0	0

^a^Presumptive presence *of Bartonella* species but species identification cannot be confirmed as *Bartonella* sequence was obtained only by *gltA*

**Table 4:** *Bartonella* prevalence and *Bartonella* species in fleas collected from rodents captured in Minnesota (June 2016) and Washington (April 2019)

Flea taxonomy	Collection state	*Bartonella* testing			*Bartonella* species		
		No. tested	No. positive	Prevalence (%)	*B. vinsonii* subsp. *arupensis*	*B. grahamii*	*B. volans*
*Ctenophthalmus pseudagrytes*	Minnesota	1	0	0			
*Megabothris* spp.	Minnesota	9	3	33.3		3	
*Opisodasys pseudarctomys*	Minnesota	1	1	100			1
*Orchopeas howardi*	Minnesota	10	1	10			1
*Orchopeas leucopus*	Minnesota	23	8	34.8	8		
*Orchopeas* spp.	Minnesota	24	12	50			12
Subtotal		68	25	36.8	8	3	14
*Catallagia spp.*	Washington	5	2	40	2		
Ceratophyllidae (Family)	Washington	1	1	100	1		
*Hystrichopsylla schefferi*	Washington	2	0	0			
*Monopsyllus wagneri*	Washington	2	1	50	1		
*Opisodasys keeni*	Washington	14	6	42.9	6		
*Opisodasys* spp.	Washington	15	9	60	9		
Others^a^	Washington	3	0	0			
Subtotal		42	19	45.2	19		

^*a*^Flea species was not identified

In Washington, blood was collected from 43 individual rodents. All rodents were *P. maniculatus* (Table 2). Of the mice, 19 were infested with ticks, and 20 were infested with fleas. A total of 56 ticks including 45 *I. pacificus* larvae and 16 *I. angustus* (11 larvae and 5 nymphs) were collected and tested (Table 3). Among the ticks, 17 (10 *I. pacificus* larvae, 5 *I. angustus* larvae, and 2 *I. angustus* nymphs) were collected from 7 *Bartonella*-infected rodents (see the following section “*Bartonella* spp. in rodents, fleas, and ticks from Washington” for data on *Bartonella*-infected rodents). A total of 42 fleas of seven species were collected and tested (Table 4).

### Bartonella* spp. in rodents, fleas, and ticks from Minnesota*

Rodents: *Bartonella* DNA was detected in 55 (25.6%) of the 215 tested rodent blood samples by at least two of the four targets (*gltA*, *ssrA*, *rpoB*, and *groEL*) used in the quadruplex PCR amplicon next-generation sequencing assay [51]. The *Bartonella*-infected rodents were distributed at all sites with prevalence ranging from 12.2% to 33.3% among sites (Table 1). *Bartonella* prevalence in rodents was similar among the five sites (χ^2^ = 8.1, df = 4, *p* = 0.09). Among rodent species, *Bartonella* prevalence was the highest in *P. maniculatus* (38.1%; 8/21) followed by *C. gapperi* (32.4%; 12/37) and *P. leucopus* (28%; 35/125). No *Bartonella* DNA was detected in other rodent species (Table 2). Sequence analysis showed the *Bartonella* detected in the *P. maniculatus* (*n* = 8) and the *P. leucopus* (*n* = 35) belonged to *B. vinsonii* subsp. *arupensis*, a species specific to *Peromyscus* mice [4, 12]. The *Bartonella* detected in the *C. gapperi* were identified as *B. grahamii* (9/12) and *B. vinsonii* subsp. *vinsonii* (3/12) (Table 2).

From the 159 tick-infested rodents, *Bartonella* DNA was detected in 37 (23.3%) of them (Table 5). The *Bartonella* prevalence in tick-infested rodents was not different compared with the prevalence in tick-free rodents (32.1%; 18/56) (χ^2^ = 1.71, *p* = 0.19), indicating ticks picked hosts randomly to feed and presence of ticks on rodents did not influence the infection status of the rodent.

**Table 5:** Comparison of *Bartonella* prevalence in tick-infested rodents and tick-free rodents

Collection state	Tick-infested rodents			Tick-free rodents		
	No. rodents	No. *Bartonella*-positive rodents	*Bartonella* prevalence (%)	No. rodents	No. *Bartonella*-positive rodents	*Bartonella* prevalence (%)
Minnesota	159	37	23.3	56	18	32.1
Washington	19	7	36.8	24	5	20.8

Fleas: *Bartonella* DNA was detected in 25 (36.8%) of the 68 tested fleas by at least two targets as stated above with variable prevalence among the flea species (0–100%) (Table 4). Sequence analysis showed that *Bartonella* detected in the fleas belonged to *B. vinsonii* subsp. *arupensis*, *B. grahamii*, and *Bartonella volans* (Table 4).

Ticks: Of all 792 *I. scapularis* ticks, including the 140 collected from *Bartonella*-positive rodents, *Bartonella* DNA was detected in only one larva by *gltA*, suggesting the presumptive presence [51] of *Bartonella* species in the tick. This larval tick was collected from a *P. leucopus* mouse from site CARI (Table 3). The *gltA* sequence showed the *Bartonella* was *B. vinsonii* subsp. *arupensis*; however, the species was not confirmed as all of the other three targets were negative.

### Bartonella* spp. in rodents, fleas, and ticks from Washington*

*Rodents* *Bartonella* DNA was detected in 12 (27.9%) of the 43 tested rodent blood samples by at least two targets. Sequence analysis identified the *Bartonella* as *B. vinsonii* subsp. *arupensis*, showing strong host specificity (Table 2).

From the 19 tick-infested rodents, *Bartonella* DNA was detected in 7 (36.8%) of them (Table 5). Similar to results from Minnesota, the *Bartonella* prevalence in tick-infested rodents was similar to that in tick-free rodents (20.8%; 5/24) (χ^2^ = 1.35, *p* = 0.25).

*Fleas* *Bartonella* DNA was detected in 19 (45.2%) of the 42 tested fleas by at least two targets with variable prevalence among the flea species (0–100%) (Table 4). Sequence analysis showed that all 19 *Bartonella* DNA detected in the fleas was *B. vinsonii* subsp. *arupensis* (Table 4).

*Ticks* no *Bartonella* DNA was detected in any of the 45 *I. pacificus* ticks (including the 10 collected from *Bartonella*-infected rodents) or the 16 *I. angustus* ticks (including the 7 collected from *Bartonella*-infected rodents) by any target.

## Discussion

We investigated *Bartonella* spp. in *Peromyscus* mice and rodents of other species captured in Minnesota and Washington and their ectoparasites, fleas and *Ixodes* ticks. Similar to other reports [1–6, 55, 56], *Bartonella* spp. were frequently detected in the rodents and their fleas, with 25.6% (Minnesota) and 27.9% (Washington) of rodents infected and 36.8% (Minnesota) and 45.2% (Washington) of fleas infected. Several *Bartonella* spp., including *B. vinsonii* subsp. *arupensis*, *B. vinsonii* subsp. *vinsonii*, *B. grahamii*, and *B. volans*, were identified in the rodents and/or their fleas. A strong host specificity was observed between *Peromyscus* mice and *B. vinsonii* subsp. *arupensis* as previously reported [4, 10], while fleas of different species may share the same *Bartonella* species. These results indicate *Bartonella* spp. are prevalent in the communities we sampled. Although vector competence of fleas has not been evaluated for these *Bartonella* species, given the high prevalence of *Bartonella* infection in the fleas, they are suspected of playing a role in the enzootic transmission of the *Bartonella* species.

By contrast, of over 850 blood-fed immature *I. scapularis*, *I. pacificus*, and *I. angustus* ticks collected from *Peromyscus* mice and other rodents, which contained 157 ticks collected from *Bartonella*-infected rodents, only one *I. scapularis* larva was presumptively positive for *Bartonella* by one target, *gltA*. When comparing tick-infested rodents and tick-free rodents, *Bartonella* prevalence was similar, suggesting that ticks picked hosts randomly to feed and tick infestation status did not influence infection status of the sampled rodents.

These findings demonstrate an extremely low acquisition rate of *Bartonella* spp. in blood-feeding *Ixodes* ticks. Our results were discordant with those reported by Rynkiewicz and others who detected *Bartonella* DNA in 13.3% of *I. scapularis* collected from *P. leucopus* mice in southern Indiana [43]. Notably, their detections were based on 16S rRNA results. 16S rRNA is a highly conserved target that shares homology with a wide range of soil bacteria. Studies utilizing non-specific targets could potentially yield falsely high prevalence of *Bartonella* infections in ticks [57, 58]. Furthermore, the presence of microbial DNA within a tick does not conclusively establish the viability of the microbe or the competency of the tick species as a vector [57]. Given the lack of *Bartonella* DNA in blood-feeding *Ixodes* spp. larvae and nymphs, we found no evidence to suggest transovarial or transstadial transmission of *Bartonella* by *Ixodes* ticks. Indeed, our results suggest that *Ixodes* ticks rarely acquire *Bartonella* spp. by blood feeding on bacteremic hosts. However, ticks examined in this study were collected at varying stages of engorgement, and bacteremia likely differed among their rodent hosts. Using these samples, we cannot determine whether acquisition rates would have been higher if ticks had been allowed to feed to repletion on highly bacteremic hosts.

Previous laboratory experiments showed that *Ixodes ricinus* can acquire *Bartonella* infection when continuously fed on blood with very high bacteremia (10^8^ – 10^9^ CFU) [59, 60]. However, such high bacterial concentrations are rarely seen in natural infections [57; 61] and may not be relevant under natural conditions. Vector competence has not been demonstrated for *I. scapularis* or *I. pacificus* and any *Bartonella* species that naturally occur in the US. Laboratory transmission studies on *I. scapularis* and *I. pacificus* are warranted to elucidate acquisition, survival, and transstadial transmission rates. Our previous study conducted over a much broader geographic range found no *Bartonella* DNA in host-seeking *Ixodes* spp. ticks [35]. Such a finding could arise from either a lack of exposure to *Bartonella*-infected hosts, failure to acquire infection through feeding on infected hosts, high mortality in *Bartonella*-infected ticks, or inefficient transstadial transmission. Here, we showed that tick-infested rodents were commonly infected with *Bartonella* sp. (most commonly *B. vinsonii* subsp. *arupensis*), but ticks feeding on infected hosts were seldom, if ever, infected with *Bartonella*. Laboratory transmission studies are needed to assess acquisition rates in *Ixodes* spp. ticks fed to repletion, and, if ticks are proven to acquire infection, assess survival rates of infected ticks and transstadial transmission efficiency from larva to nymph and nymph to adult.

## Conclusions

The high prevalence of *Bartonella* spp. in rodents and rodent-associated fleas coupled with extremely low prevalence of *Bartonella* spp. in blood-fed ticks suggests that although *Ixodes* spp. ticks commonly encounter *Bartonella* spp. in rodents, they rarely acquire infection through blood feeding. Laboratory transmission studies are needed to assess acquisition rates in fully engorged ticks and to assess transstadial transmission efficiency if ticks acquire *Bartonella* infections from feeding to repletion. *Bartonella vinsonii* subsp. *arupensis* would be a good candidate for transmission study based on the common association between the *Bartonella* species and *Peromyscus* sp. mice that *Ixodes scapularis* and *I. pacificus* ticks commonly feed on.

## Data Availability

No datasets were generated or analysed during the current study.
